# Innovations in the Delivery of Bioactive Compounds for Cancer Prevention and Therapy: Advances, Challenges, and Future Perspectives

**DOI:** 10.3390/ph19010060

**Published:** 2025-12-27

**Authors:** Carlos A. Ligarda-Samanez, Mary L. Huamán-Carrión, Jackson M’coy Romero Plasencia, Dante Fermín Calderón Huamaní, Bacilia Vivanco Garfias, Jenny C. Muñoz-Saenz, Maria Magdalena Bautista Gómez, Jaime A. Martinez-Hernandez, Wilber Cesar Calsina-Ponce

**Affiliations:** 1 Nutraceuticals and Biomaterials Research Group, Universidad Nacional José María Arguedas, Andahuaylas 03701, Peru; 2 Department of Mathematics and Physics, Universidad Nacional de San Cristóbal de Huamanga, Ayacucho 05000, Peru; jackson.romero@unsch.edu.pe; 3 Department of Environmental Engineering, Universidad Nacional San Luis Gonzaga, Ica 11001, Peru; dante.calderon@unica.edu.pe (D.F.C.H.); jaime.martinez@unica.edu.pe (J.A.M.-H.); 4 Department of Obstetrics, Universidad Nacional de San Cristóbal de Huamanga, Ayacucho 05000, Peru; bacilia.vivanco@unsch.edu.pe (B.V.G.); maria.bautista@unsch.edu.pe (M.M.B.G.); 5 Environmental Engineering School, Universidad Continental, Huancayo 12006, Peru; jmunoz@continental.edu.pe; 6 Social Sciences Faculty, Universidad Nacional del Altiplano, Puno 21001, Peru; wcalsina@unap.edu.pe

**Keywords:** drug delivery, nanoformulations, tumor microenvironment, pharmacokinetics, clinical translation, bioavailability

## Abstract

Naturally occurring bioactive compounds represent a promising option for cancer prevention and therapy due to their ability to modulate apoptosis, angiogenesis, inflammation, oxidative stress, and cell signaling. However, their clinical impact is limited by low bioavailability, chemical instability, rapid metabolism, and poor tumor microenvironment accumulation. Innovative delivery platforms, including lipid and polymeric nanoparticles, liposomes, micelles, nanoemulsions, hydrogels, and stimulus-responsive systems, have been developed to improve stability, absorption, tumor specificity, and therapeutic efficacy. This review integrates molecular mechanisms, preclinical and clinical evidence, and recent technological advances, highlighting both potential and limitations. Although several compounds show encouraging results in cell and animal models, only a small number have progressed to early clinical trials, where outcomes remain heterogeneous and often fail to replicate preclinical magnitudes. Regulatory barriers, a lack of formulation standardization, and the absence of predictive biomarkers persist. Sustainability is also addressed through the valorization of agrifood by-products and green extraction processes. This review provides an integrative framework linking molecular mechanisms, advanced delivery technologies, clinical translation, and sustainability, offering a broader perspective than conventional reviews. Future perspectives emphasize multicenter trials, comparative designs, and the development of regulatory guidelines for nanoformulated bioactive compounds.

## 1. Introduction

Cancer continues to be one of the leading causes of death worldwide. The most recent epidemiological reports show a steady increase in both incidence and mortality, with marked variations across regions, age groups, and access to diagnosis and treatment [1,2,3]. This situation poses a significant health and social challenge, as the disease not only compromises the health of millions of people but also has a significant economic impact. In addition to the direct costs of care, there is the so-called “financial toxicity,” which affects households and reduces labor productivity, factors that together increase pressure on health systems and deepen existing inequalities [4,5,6].

On the other hand, conventional oncology treatments, such as chemotherapy, radiotherapy, and immunotherapy, have documented limitations that limit their effectiveness. These include primary and acquired tumor resistance, disease recurrence, and toxicity profiles that affect patients’ quality of life, decrease adherence, and compromise clinical outcomes [7,8,9]. These limitations have spurred the search for innovative approaches that complement or enhance current therapies, with an emphasis on less-toxic, more-selective strategies.

In this context, naturally occurring bioactive compounds have gained prominence in cancer prevention and therapy. Among them are polyphenols, carotenoids, alkaloids, and terpenes, known for modulating critical stages of carcinogenesis and acting as adjuvants through proapoptotic, antiangiogenic, antimetastatic, epigenetic, and immunomodulatory effects, among others [10,11,12,13]. Representative examples are curcumin, resveratrol, epigallocatechin gallate (EGCG), and lycopene, which have been extensively studied in preclinical and clinical models. However, their low bioavailability, chemical instability, and rapid metabolism limit their efficacy. Therefore, in recent years, innovative delivery systems, such as liposomes, polymeric nanoparticles, and nanoemulsions, have been developed to improve stability, absorption, and tumor selectivity [11,12,13,14].

Despite these advances, significant controversies remain. Several polyphenols exhibit dual redox behavior: depending on the dose and context, they can act as protective antioxidants or as pro-oxidants with antitumor potential. This ambivalence explains some of the discrepancies observed between in vitro studies, in vivo trials, and clinical trials [15,16]. Furthermore, these variations depend on multiple factors, including concentration and exposure time, formulation and vehicle used, individual metabolism, gut microbiota, and the tumor microenvironment. In this regard, it is essential to standardize experimental designs and reporting to achieve more rigorous comparisons and build stronger consensus [12,14,17]. However, the breadth of existing studies, most reviews address these aspects in isolation. Current literature rarely integrates molecular mechanisms, delivery innovations, biological limitations, clinical translation, and sustainability within a single framework. This lack of synthesis prevents a comprehensive understanding of how these components interact and limits the development of more effective and scalable therapeutic strategies.

Likewise, the available literature reveals significant gaps. Most reviews maintain a descriptive approach and fail to integrate molecular mechanisms with delivery platforms or clinical outcomes in a critical way. Similarly, few evaluations report metrics on the quality, safety, and traceability of formulations [10,12]. There is also limited literature linking innovations in delivery systems to scalability and sustainability. Given that agri-food by-products represent an alternative source of bioactive compounds and that green extraction processes offer environmental and economic advantages, it is essential to connect these perspectives with the design of innovative systems and their eventual clinical and regulatory translation [18,19]. Moreover, the persistent disconnect between promising preclinical results and modest or inconsistent clinical outcomes highlights the need for stronger translational approaches. Incorporating standardized methodologies, predictive biomarkers, clinically relevant dosing strategies, and regulatory considerations is essential to bridge laboratory innovation with real therapeutic impact.

Within this framework, this review article aims to offer a critical and forward-looking summary of innovations in the delivery of bioactive compounds applied to cancer prevention and therapy. To this end, the following objectives are set out:

- Integrate mechanistic evidence of bioactive compounds relevant to oncology.
- Analyze recent advances in delivery systems, including nano- and microstructures, liposomes, hydrogels, and stimulus-responsive platforms.
- Coordinate the transition from laboratory to clinical practice under quality and safety standards.
- Incorporate the dimension of sustainability, considering alternative sources, green extraction processes, and the valorization of by-products.

**Figure 1:**
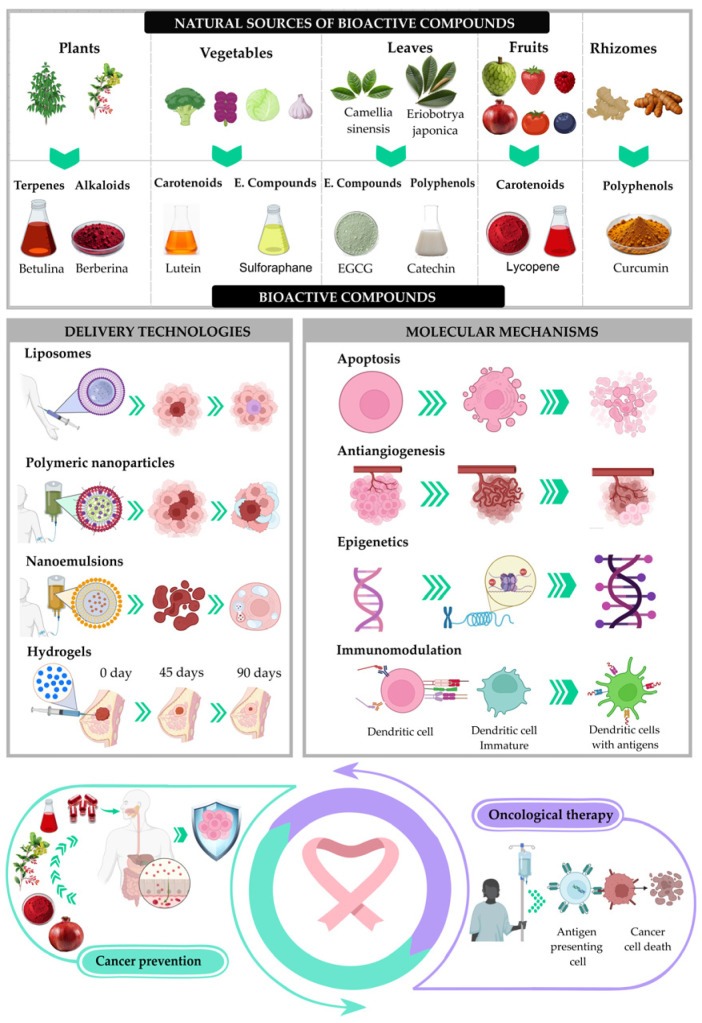
Integrative concept map illustrating the relationships among sources of bioactive compounds, major chemical groups, emerging delivery technologies, molecular mechanisms involved, and their applications in cancer prevention and therapy.

This approach seeks to link prevention, treatment, technological innovation, and sustainability, and to outline the main challenges and prospects in a field of growing scientific and social relevance. Figure 1 presents an integrative conceptual map summarizing the relationships among sources of bioactive compounds, the main chemical groups identified, emerging delivery technologies to overcome their limitations, the molecular mechanisms involved, and, finally, their impact on cancer prevention and therapy. To systematically address these challenges, this review synthesizes the current evidence on the molecular mechanisms, physicochemical limitations, innovative delivery platforms, and translational advances of key bioactive compounds, providing an integrated framework that guides their potential application in cancer prevention and therapy. This conceptual figure was constructed by synthesizing recurring themes identified across the analyzed literature, allowing for a visual representation of the integrated framework proposed in this review.

## 2. Review Methodology

This narrative review follows a structured approach and uses a structured bibliographic search methodology [20,21] to provide a clear, up-to-date, and critical overview of recent advances in the delivery of bioactive compounds for cancer prevention and treatment. The review was not designed as a systematic or scoping review, but rather as a critical narrative synthesis of the current state of the art. Searches were conducted in the PubMed, Scopus, Web of Science, and ClinicalTrials.gov databases, supplemented by queries across MDPI, Elsevier, SpringerLink, Taylor & Francis, and Wiley, and limited to articles published preferably between 2020 and 2025.

The search strategy included keywords such as bioactive compounds, cancer prevention, cancer therapy, phytochemicals, drug delivery, nanoparticles, encapsulation, and clinical trials. No formal reporting guidelines such as PRISMA or PRISMA-ScR were applied, given the narrative nature of the review; however, general principles of clarity, transparency, and critical appraisal commonly recommended for narrative reviews were considered. After removing duplicate records and reviewing titles and abstracts, inclusion criteria were applied, prioritizing thematic relevance, methodological rigor, and relevance to oncology and pharmaceutical nanotechnology.

Studies that were not directly related to cancer or delivery systems, those with methodological deficiencies or without verifiable data, and those focused exclusively on general antioxidant activity were excluded. A total of 260 articles were selected for detailed analysis and critical synthesis, covering in vitro and in vivo studies, clinical trials, and recent reviews. This selection allowed for a basic stratification of evidence levels, facilitating a comparative assessment of the translational maturity of the included studies. The information obtained was organized and analyzed comparatively, with a critical approach, to identify advances, limitations, and knowledge gaps relevant to future research.

## 3. Bioactive Compounds with Potential in Cancer Prevention and Therapy

The bioactive compounds present in plants, fruits, vegetables, and other natural organisms have attracted considerable interest due to their ability to prevent or slow the development of cancer. These compounds belong to different chemical groups, including polyphenols, carotenoids, terpenes, alkaloids, and other emerging compounds, which can act on various processes involved in tumor onset and progression, helping to block or reverse these mechanisms.

Among the most important actions are the activation of cancer cell death (apoptosis), the reduction in the formation of new blood vessels that feed the tumor (angiogenesis), the arrest of uncontrolled cell growth, and the regulation of genes and signals that influence the tumor environment and the body’s response [22,23,24]. However, despite encouraging results from preclinical in vitro and in vivo studies, the therapeutic application of these compounds remains limited by issues of bioavailability, physicochemical stability, and poor clinical translation into human trials [25,26]. Despite the wide range of biological actions attributed to these compounds, the therapeutic effects observed in clinical studies remain limited. Although polyphenols, terpenes, alkaloids, and carotenoids influence apoptosis, angiogenesis, inflammation, and other central processes in tumor development, these mechanisms rarely lead to meaningful clinical improvements. Most early-phase trials report modest or inconsistent changes in surrogate biomarkers, and the impact on tumor progression, recurrence, or patient survival is usually minimal. This persistent gap between mechanistic promise and clinical performance highlights the difficulty of translating preclinical efficacy into real therapeutic benefit. It emphasizes the need for research approaches that connect biological mechanisms with pharmacokinetics, pharmacodynamics, and clinically relevant outcomes.

In addition, the biological activity of these compounds is strongly influenced by interindividual variability related to metabolism, gut microbiota composition, and genetic polymorphisms involved in absorption and detoxification pathways. These factors can alter circulating concentrations and therapeutic responses, partly explaining the heterogeneous outcomes of clinical trials. Establishing standardized analytical methods, dose–response relationships, and clinically relevant biomarkers is therefore essential to improve comparability among studies and strengthen the clinical translation of bioactive compounds. Table 1 below summarizes the main bioactive compounds included in this review, their natural sources, mechanisms of action, available evidence, and known limitations.

Taken together, the bioactive compounds described exhibit a wide range of molecular mechanisms that can intervene at key stages of tumor development. Their antioxidant, anti-inflammatory, proapoptotic, and antiangiogenic activities, combined with modulation of signaling pathways involved in cell cycle regulation and metastasis, support the growing interest in their study as preventive agents or adjuvants in cancer therapy. However, the evidence also highlights important limitations, particularly regarding bioavailability, metabolic variability, potential drug interactions, and the limited standardization of effective doses in humans. Therefore, although preclinical results are promising, there is a need to advance to better-designed clinical studies to determine their true efficacy, safety, and potential integration into complementary therapeutic strategies. This synthesis reinforces the need to continue investigating both their biological activities and the technological approaches that may optimize their stability, absorption, and clinical use.

## 4. Innovative Platforms for the Delivery of Bioactive Compounds

The design of nanostructured delivery systems has become a key tool for improving the therapeutic performance of bioactive compounds used in cancer prevention and treatment [11,12,13,14]. Most of these molecules have intrinsic limitations such as low solubility, instability to oxidation or pH changes, accelerated metabolism, and limited accumulation in the tumor microenvironment, which restrict their clinical efficacy [10,11,12,13,14]. In response, platforms have been developed that can protect compounds during their biological transit, modulate their release, and direct them to specific tissues, thereby optimizing their absorption, bioavailability, and antitumor activity [11,12,13,14].

These technologies include lipid and polymeric nanoparticles, liposomes, micelles, nanoemulsions, hydrogels, stimulus-responsive systems, as well as hybrid platforms and emerging approaches based on self-assembly or self-emulsification. Although they all share the purpose of improving the transport and stability of bioactives, they differ in key aspects such as composition, internal architecture, encapsulation mechanism, loading capacity, release kinetics, and the degree of experimental or clinical advancement [11,12,13,14].

Each platform offers particular advantages. Lipid and polymeric nanoparticles stand out for their versatility in encapsulating polyphenols, terpenes, and alkaloids; liposomes are considered mature, biocompatible technologies; micelles and nanoemulsions facilitate the solubilization of highly hydrophobic compounds; and hydrogels or stimulus-responsive systems allow for localized release under specific tumor conditions. Hybrid systems and emerging platforms seek to integrate complementary properties to maximize stability and selectivity [11,12,13,14]. A key mechanistic rationale for these delivery systems is their ability to overcome the intrinsic physicochemical limitations of bioactive compounds, such as low solubility, chemical instability, poor membrane permeability, and rapid metabolism, thereby restricting systemic exposure. These constraints justify the use of platforms that enhance dissolution (nanoemulsions and micelles), protect labile compounds during circulation (liposomes or coacervates), improve cellular uptake (surface-engineered nanoparticles), or regulate release kinetics (polymeric matrices). Each platform targets a specific mechanistic barrier, aligning molecular stability, bioavailability, and pharmacokinetics with oncology therapeutic goals. Given this technological heterogeneity, it is necessary to compare their fundamental characteristics and levels of validation critically. Table 2 summarizes these aspects, contrasting the types of platforms, the compounds evaluated, the delivery mechanisms, their advantages, technical limitations, and the current state of preclinical and clinical evidence.

In the relationship between the bioactive compound and the delivery platform, the selection of the most appropriate system should primarily consider the bioactive compound’s physicochemical properties, such as solubility and lipophilicity profiles and stability, as well as the intended therapeutic objective. In the case of markedly hydrophobic molecules, often characterized by high lipophilicity and low aqueous solubility, polymeric micelles and nanoemulsions are advantageous alternatives because they can incorporate the bioactive compound into hydrophobic lipid domains, thereby facilitating solubilization and transport in aqueous systems [179,180,181,182,183,184]. By contrast, when greater encapsulation versatility and flexibility in system design are required, for example, to tailor release profiles, functionalize surfaces, or adapt to different classes of compounds, polymeric nanoparticles based on biodegradable polymers stand out as robust and highly modulable platforms [167,168,169,170,171,172,173]. Likewise, in clinical scenarios where localized administration, retention at the site of action, and sustained release are prioritized, as is often the case for sensitive compounds or local therapeutic strategies, hydrogels and, at a more advanced stage of development, stimuli-responsive systems sensitive to pH, temperature, redox state, or external stimuli provide more precise spatial and temporal control, albeit at the cost of increased design complexity and scalability challenges [193,194,195,196,197,198,199,200].

Overall, the comparison shows that no single platform can overcome all the limitations associated with bioactive compounds. Each technology offers specific advantages, but also has restrictions that must be considered depending on the chemical structure of the bioactive compound, its solubility profile, the route of administration, and the therapeutic purpose. Lipid and polymeric nanoparticles remain the most versatile; liposomes maintain a strong position due to their safety record; micelles and nanoemulsions are particularly suitable for highly hydrophobic molecules; and hydrogels and stimulus-responsive systems represent promising alternatives for localized delivery. Hybrid systems and emerging platforms, while attractive, require further standardization, reproducibility, and clinical validation before widespread implementation. These contrasts reinforce the need for formulation approaches tailored to each compound and each oncology indication, as well as the development of multimodal strategies that integrate multiple technologies to maximize efficacy and safety.

Beyond predominantly passive nanocarriers, an emerging and genuinely innovative direction in the field of drug delivery involves active micro- and nanomotors and microrobots capable of self-propulsion or external actuation. These systems may operate through chemical or biochemical reactions or be controlled by external physical fields, such as magnetic fields or acoustic stimuli, enabling active navigation, enhanced tissue penetration, and more localized delivery of therapeutic agents compared with passive platforms, which are limited by diffusion and systemic circulation [215,216,217,218]. In oncology-oriented applications, micro- and nanomotor-based concepts have been proposed as strategies to overcome some of the limitations of conventional nanomedicine, including insufficient intratumoral penetration and heterogeneous drug distribution, by introducing controlled motion and spatiotemporal guidance [218]. Nevertheless, despite their high innovative potential, these active systems remain primarily at the proof-of-concept and preclinical evaluation stages, and their clinical translation will require substantial advances in areas such as biosafety and biodegradability, in vivo control and tracking, scalability of manufacturing processes, and regulatory standardization [215,216,217,218].

## 5. Clinical and Translational Evidence

The clinical and translational evidence on bioactive compounds in oncology presents a heterogeneous landscape, encompassing diverse chemical groups, including polyphenols, carotenoids, terpenes, alkaloids, and emerging compounds, and evaluated across a wide range of cancer types and at various stages of drug development. According to the recent literature, most of these compounds remain in preclinical phases, involving cell-based and animal model studies, while only a limited number have advanced to early clinical stages, mainly Phase I and Phase II, with variable results in tumors such as breast, prostate, colon, lung, and skin cancers [10,12,25]. This uneven distribution reflects both the therapeutic potential demonstrated in experimental models and the barriers that have hindered their progression toward more robust clinical trials.

Within the field of cancer prevention, some bioactive compounds show more consistent evidence when evaluated in prevalent tumors such as colorectal, prostate, esophageal, and breast cancers. Curcumin, for example, has demonstrated beneficial effects in colorectal cancer prevention through epigenetic modulation and inflammation reduction in early clinical studies, although results vary depending on formulation and administered dose [34,36]. Lycopene has also been widely studied in prostate and gastrointestinal cancers, with meta-analyses supporting its protective association when consumed consistently either through diet or improved formulations [57,58,59,60]. Another notable compound is EGCG, which has been explored in preventive settings for prostate and esophageal cancer, showing reductions in tumor progression markers in controlled trials. However, limitations persist due to low bioavailability and interindividual metabolic variability [13,140,141,142]. Together, these studies reveal significant preventive potential, although the evidence remains insufficient to support broad clinical recommendations because of the lack of standardization in concentrations, duration, and administration methods.

In the therapeutic setting, several bioactive compounds have been evaluated as adjuvants in breast, prostate, lung, colon, and bladder cancers. Resveratrol stands out for its anti-inflammatory, proapoptotic, and mitochondrial signaling-modulating effects, with early clinical studies reporting modest reductions in tumor markers in breast and prostate cancer. However, its overall therapeutic impact remains limited due to rapid metabolism and low stability in vivo [160,165]. Quercetin has also shown promising results as a coadjuvant, particularly in bladder cancer and hormone-dependent tumors, where advanced formulations, including lipid nanoparticles and intravesical gels, have improved local delivery and cytotoxicity [159,168]. Despite these advances, most clinical trials continue to yield mixed results, with variability attributed to interindividual metabolic differences, inconsistent standardization of evaluated formulations, and heterogeneous dosing. These limitations highlight the need for pharmacokinetic optimization strategies and more rigorous clinical trial designs to translate the therapeutic potential observed in preclinical models effectively.

Despite growing interest in the use of bioactive compounds for cancer prevention and treatment, their progression into advanced clinical stages remains limited, with a predominance of preclinical studies and minimal transition into Phase III or Phase IV trials. Among the main challenges is the lack of standardization in evaluated formulations, which complicates comparison across studies and prevents the establishment of reproducible therapeutic doses [25,159]. Pronounced interindividual metabolic variability, driven by factors such as genetic differences, gut microbiota composition, and dietary habits, also affects the bioavailability and clinical consistency of molecules such as polyphenols, carotenoids, and terpenes, thereby limiting their therapeutic impact in real-world settings [140,160]. In addition, the absence of predictive biomarkers of response restricts the identification of patient subgroups most likely to benefit from these interventions, slowing their integration into larger clinical trials. These factors explain the gap between the strong performance observed in experimental models and the modest effects recorded in clinical studies, underscoring the need for more standardized strategies, personalized medicine approaches, and biomolecular validation before these compounds can be consolidated as therapeutic tools.

It is essential to clearly distinguish the different types of clinical studies conducted to date, as their conclusions vary considerably depending on methodological rigor. Observational studies frequently report associations between higher dietary intake of polyphenols, carotenoids, or terpenes and a lower risk of several cancers. However, these findings cannot be interpreted as causal because they are strongly influenced by diet, metabolic differences, and lifestyle factors that are difficult to control. More solid information comes from interventional studies, mainly Phase I and Phase II clinical trials, yet their results remain inconsistent. Some studies report modest improvements in inflammatory or oxidative stress markers or in indicators such as prostate-specific antigen.

In contrast, others show no meaningful effects on tumor progression, recurrence, or survival. In many cases involving compounds such as curcumin, EGCG, or resveratrol, the absence of significant clinical benefits has been attributed to insufficient dosing, rapid metabolism, or low systemic availability. Understanding these differences in study design helps clarify the current clinical landscape. It underscores the need for larger, well-standardized, and mechanistically informed trials better to assess the true therapeutic potential of these bioactive molecules.

A further barrier limiting clinical translation is the insufficient characterization of toxicity, safety, and regulatory readiness for most bioactive compounds and their formulations. Although many phytochemicals are generally regarded as safe in dietary contexts, their behavior changes substantially when administered at pharmacological doses or encapsulated in nanostructured systems. Issues such as nanoparticle-induced oxidative stress, off-target accumulation, immunogenicity, and long-term biopersistence remain poorly defined in current studies. Moreover, the absence of standardized safety assays, GMP-compliant manufacturing protocols, and well-established regulatory pathways for complex delivery systems poses additional obstacles to advancing these compounds into late-stage clinical trials. These gaps highlight the need for rigorous toxicological profiling and early engagement with regulatory frameworks to ensure that promising experimental results can be translated into safe and clinically viable oncology interventions. Table 3 shows how these bioactive compounds are associated with different types of cancer and the available preclinical and early clinical evidence.

## 6. Challenges and Opportunities

Bioactive compounds face significant challenges that have limited their progress toward more established clinical stages. Among the most relevant obstacles are their low bioavailability and rapid metabolism, characteristics widely documented for polyphenols, terpenes, and carotenoids, which reduce their systemic half-life and make it difficult to achieve stable therapeutic concentrations [140,160]. Likewise, the marked variability in natural sources, associated with genetic differences, climate, post-harvest practices, and phytochemical composition, complicates the standardization of extracts and affects the reproducibility of results [25]. Added to this is the limited industrial scalability of advanced delivery technologies, such as nanoencapsulation, hydrogels, and lipid-polymer hybrid systems, whose processes require specialized equipment and high operating costs [159]. Finally, regulatory obstacles also pose a challenge: while nutraceuticals are often approved under more flexible frameworks, nanopharmaceuticals derived from natural compounds must meet strict safety, efficacy, and reproducibility requirements, which slows their transition to clinical applications [165].

Another critical challenge is the lack of methodological standardization across studies evaluating nanoformulated bioactive compounds. Variations in pH conditions, particle size determination, polydispersity index (PDI), zeta potential measurements, and encapsulation efficiency protocols hinder the comparability of results and limit the reproducibility of reported formulations. Moreover, the use of different analytical instruments, dispersants, and sample-preparation procedures frequently leads to discrepancies in stability profiles and release kinetics. Establishing harmonized characterization frameworks, aligned with international standards for nanomaterials, would substantially improve the reliability of preclinical data and facilitate regulatory assessment and clinical translation.

Clinically, biological variability and extract heterogeneity introduce additional barriers. Despite these limitations, significant opportunities are emerging that strengthen the position of bioactive compounds in oncology. One of the most notable is the use of agri-food by-products, in line with the principles of the circular bioeconomy, which enables the production of functional compounds in a sustainable manner with reduced environmental impact [18,19,21]. At the same time, there is growing interest in integrating these compounds with immunotherapy and targeted therapies, given their potential to modulate inflammatory, epigenetic, and mitochondrial pathways in a manner complementary to conventional drugs [160]. Likewise, integrating omics technologies, including genomics, transcriptomics, and metabolomics, with artificial intelligence tools enables the identification of biomarkers, the optimization of formulations, and the prediction of clinical responses with greater precision [25]. Taken together, these opportunities outline a promising scenario for the innovative application of bioactive compounds within contemporary precision oncology.

Beyond these opportunities, several forward-looking directions are emerging that could accelerate the translation of bioactive compounds into clinical practice. These include the use of AI-driven predictive models to optimize encapsulation efficiency and release profiles, surface-engineered nanocarriers designed to improve targeting of the tumor microenvironment, and the development of computational tools to model dose–response relationships and interindividual variability. Additionally, incorporating digital biomarkers and adaptive clinical trial designs may help refine patient selection and enhance the evaluation of nanoformulated bioactives in real-world settings.

**Figure 2:**
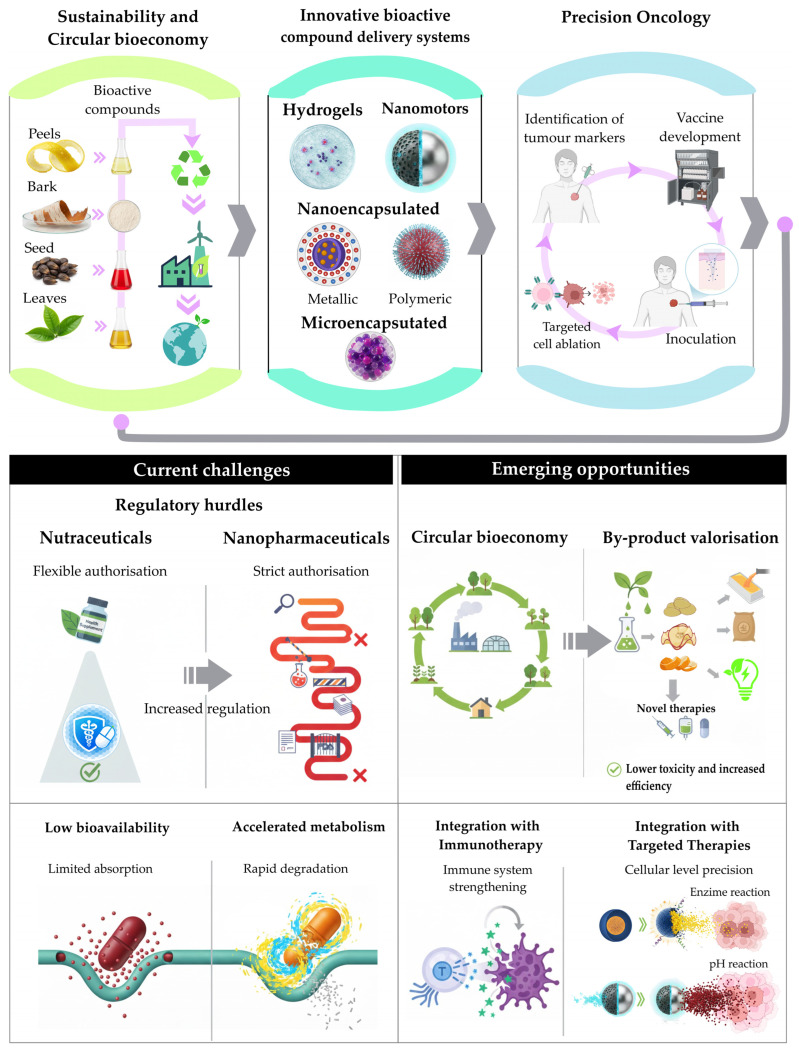
Integrative model of sustainability, innovative delivery systems, and precision oncology.

Figure 2 visually summarizes this duality between current challenges and emerging opportunities, integrating aspects of sustainability, advanced delivery technologies, and therapeutic innovation.

## 7. Future Perspectives

The clinical advancement of bioactive compounds in oncology will require coordinated efforts to overcome current limitations and consolidate their therapeutic applications. An immediate priority is the implementation of multicenter clinical trials, particularly those involving nanoformulated bioactives, to obtain more robust, generalizable data on efficacy and safety across diverse populations.

Likewise, validating efficacy biomarkers will be essential in both preventive and therapeutic contexts. The identification of molecular, metabolomic, or immunological markers will enable the selection of more responsive patient subgroups and facilitate progress toward personalized medicine strategies.

Another key direction involves comparative studies between bioactive compounds and conventional drugs, or between bioactive–drug combinations. These analyses will help elucidate potential synergies, optimize therapeutic regimens, and justify their integration into standardized clinical protocols.

Finally, the field requires the development of specific regulatory guidelines for bioactive compound delivery technologies, particularly nanoformulations, hydrogels, and hybrid systems. Such frameworks would support the harmonization of quality, reproducibility, and safety criteria, thereby accelerating their transition into advanced clinical stages and eventual therapeutic consolidation.

Emerging technologies will further shape the future of this field. Artificial-intelligence algorithms may assist in predicting encapsulation behavior, optimizing formulation parameters, and modeling complex pharmacokinetic–pharmacodynamic interactions. Surface-engineered nanocarriers represent another promising avenue to enhance tumor specificity and microenvironment responsiveness. Likewise, computational tools enabling virtual screening, dose optimization, and patient stratification modeling could substantially improve the design and success of early-phase clinical trials.

**Figure 3:**
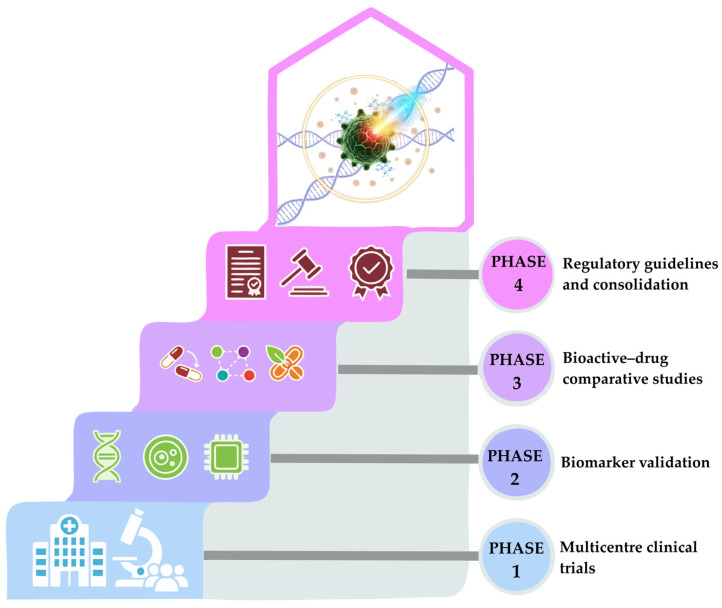
Future perspectives for the delivery of bioactive compounds and their application in cancer prevention and therapy.

Figure 3 summarizes these progressive stages, from initial trials to the establishment of specific regulatory frameworks.

## 8. Conclusions

Bioactive compounds represent one of the most promising frontiers in cancer prevention and therapy, as they can modulate multiple pathways involved in carcinogenesis. However, their clinical impact remains limited due to intrinsic factors, including low bioavailability, chemical instability, rapid metabolism, and poor tumor microenvironment accumulation. Innovative delivery platforms, including lipid and polymeric nanoparticles, liposomes, micelles, nanoemulsions, hydrogels, stimuli-responsive systems, and hybrid approaches, have demonstrated significant progress in improving the stability, absorption, selectivity, and efficacy of these compounds. However, no single technology fully overcomes all challenges.

Despite encouraging results in in vitro and in vivo studies, clinical translation remains restricted, with early-phase trials predominating and outcomes varying widely. This gap highlights the need to standardize formulations, optimize pharmacokinetic parameters, identify response biomarkers, and design more robust multicenter clinical trials to validate their actual effectiveness across diverse populations.

A critical priority moving forward is the harmonization of analytical and characterization protocols, particularly for nanoformulated bioactive systems, where disparities in particle size measurements, PDI values, zeta potential, and encapsulation efficiency methods hinder cross-study comparability. Establishing unified methodological standards aligned with international nanomaterials guidelines will strengthen reproducibility, improve regulatory assessment, and accelerate the transition of bioactive compounds toward clinically meaningful applications.

Sustainability has also emerged as a key component. The valorization of agri-food by-products and the use of green extraction processes offer opportunities to obtain bioactive compounds in an efficient, economical, and environmentally responsible manner. The integration of omics technologies and artificial intelligence will facilitate progress toward personalized formulations, the identification of response profiles, and the enhancement of synergies between natural compounds and conventional therapies.

It is also essential to emphasize that bioactive compounds should not be considered substitutes for established oncological treatments. Instead, they hold greater potential as complementary or adjuvant agents that can enhance therapeutic responses, reduce adverse effects, or improve patient-specific outcomes when integrated with conventional therapies. Clarifying this distinction is critical to ensure their appropriate clinical positioning and to guide future research toward realistic, evidence-based applications.

Overall, technological advances, the development of specific regulatory frameworks, and the integration of sustainability, innovation, and personalized medicine outline a favorable scenario for bioactive compounds to evolve from potential complementary therapies into more effective and standardized clinical interventions. The current challenge is not only to optimize their delivery but also to translate their biological complexity into safe, reproducible, and clinically meaningful applications within contemporary oncology.

## Figures and Tables

**Table 1:** Molecular mechanisms of action of the main bioactive compounds in cancer prevention and therapy.

Group of Compounds	Representative Examples	Common Natural Sources	Molecular Mechanisms of Action	Scientific Evidence and Oncological Applications	Criticisms and Limitations	Reference
Polyphenols	Ellagic acid	Strawberry, *Rubus idaeus* L., blackberry, *Fragaria vesca* L., pomegranate, walnuts, almonds, hazelnuts, flaxseed, chia, loquat, *Quercus* spp., *Eucalyptus* spp., *Castanea* spp.	Inhibition of cell proliferation and angiogenesis through the PI3K/Akt pathway; induction of apoptosis; modulation of gene expression and cell signaling.	Colon, breast, liver, prostate, pancreas, stomach, lung, endometrial, ovarian, and skin cancers.	Low bioavailability, which may limit efficacy; no established optimal dose or treatment duration in humans.	[27,28,29]
	Catechins	Green tea, cocoa, dark chocolate, apples, blueberries, blackberries, raspberries, strawberries, pecans, and hazelnuts.	Inhibition of cell proliferation through the PI3K/Akt pathway. Induction of apoptosis. Modulation of signal transduction pathways, including NF-κB inhibition and reduction in inflammation. Inhibition of angiogenesis. Modulation of oxidative stress.	Breast, prostate, colorectal, lung, esophageal, gastric, and liver cancers.	Low bioavailability due to rapid metabolism and elimination. Effective dose is not established in humans. Possible interaction with anticoagulants.	[30,31,32,33]
	Curcumin	*Curcuma longa*	Inhibition of angiogenesis. Inhibition of cancer cell proliferation. Induction of apoptosis. Antioxidant activity. Modulation of NF-κB and other pathways associated with inflammation and cell survival.	Breast, colorectal, gastric, pancreatic, ovarian, lung, prostate, esophageal, and endometrial cancers.	Prolonged consumption may cause hepatic alterations. May exert anticoagulant effects. May cause gastrointestinal discomfort. Low bioavailability, which limits its clinical efficacy.	[34,35,36,37]
	Quercetin	Onion, dark cocoa, elderberries, cranberries, capers, apples, asparagus, cabbage, broccoli, oregano, fennel, watercress, *Fagopyrum esculentum*, *Opuntia stricta*, pear, grape, and cherry.	Antioxidant activity. Anti-inflammatory effects. Induction of apoptosis.	Breast, prostate, colon, lung, liver, nasopharyngeal, kidney, pancreatic, ovarian, brain, and oral cavity cancers.	Variable bioavailability, influenced by the food matrix and metabolism. No consensus on the optimal dose required to achieve clinical effects in humans.	[38,39,40,41,42,43]
	Resveratrol	Grape skin, red wine, strawberries, blueberries, blackberries, walnuts, almonds, peanuts, pistachios, and chocolate.	Inhibition of cancer cell proliferation. Antioxidant activity. Modulation of gene expression. Induction of apoptosis. Regulation of inflammation.	Breast, prostate, lung, colorectal, pancreatic, liver, ovarian, and endometrial cancers.	Low bioavailability due to rapid metabolism and elimination. Limited pharmacokinetics, with low effective plasma concentrations. Possible nephrotoxicity at high doses. Drug interactions, especially with anticoagulants, which may increase the risk of bleeding.	[44,45,46,47]
	Hesperidin	*Citrus sinensis*, *Citrus limon*, lime, grapefruit, mint.	Inhibition of cell proliferation. Induction of apoptosis. Early cell cycle arrest. Antioxidant activity. Modulation of signaling pathways.	Breast cancer.	Low water solubility. Limited bioavailability. Rapid systemic excretion. High affinity for plasma proteins. Susceptibility to degradation caused by light, oxygen, and temperature fluctuations. A mandatory requirement for its clinical viability is the development of complex formulations that enhance its stability and bioavailability.	[48]
	Naringenin	Grapefruit, orange.	Inhibition of cell proliferation. Induction of apoptosis. Modulation of autophagy. Autophagy modulation through gene regulation of ATG5 and LC3. Disruption of DNA–protein interactions. Inhibition of polyamine synthesis.	Liver, breast, gastric, cervical, pancreatic, colon, and hematological cancers.	Low systemic bioavailability. Suboptimal pharmacokinetics at tumor sites. Low water solubility. Insufficient hepatic retention. A mandatory requirement for clinical viability is the development of complex formulations.	[49]
	Eriodictyol	*Eriodictyon californicum*, lemon, lime.	Induction of ferroptosis. BACH1 stabilization. GPX4 repression. GSH (reduced glutathione) depletion.	Bone cancer.	Low bioavailability and rapid metabolism. Hydrophobic nature and limited systemic distribution. Short biological half-life. Requirement for nanocarriers to enable tumor-targeted delivery. Risk of non-specific distribution. Limited clinical data on toxicity and therapeutic window.	[50]
	Didymin	Orange and lemon.	Modulation of the PI3K/Akt pathway. Induction of apoptosis. Inhibition of cell migration and invasion. Inhibition of cell proliferation.	Gastric cancer and neuroblastoma.	Low bioavailability and rapid metabolism. Hydrophobic nature and limited systemic distribution. Short biological half-life. Risk of non-specific distribution. Limited clinical evidence. Requirement for advanced delivery systems.	[51]
	Poncirin	*Poncirus trifoliata*.	Induction of apoptosis. Inhibition of cancer cell viability. Inhibition of cell proliferation.	Cervical and breast cancers.	Low bioavailability. Limited clinical evidence, as current findings are largely restricted to in vitro models. Short biological half-life, requiring sustained delivery systems or high doses to maintain therapeutic effects. A mandatory requirement for clinical viability is the development of complex formulations.	[52]
	α-mangostin	*Garcinia mangostana* L.	Induction of apoptosis. Modulation of autophagy. Inhibition of cell proliferation via the PI3K/Akt pathway.	Breast cancer.	Low bioavailability due to rapid metabolism and elimination. Limited pharmacokinetics, with low effective plasma concentrations. Possible nephrotoxicity at high doses. Drug interactions, especially with anticoagulants. A mandatory requirement for clinical viability is the development of complex formulations.	[53]
	γ-mangostin	*Garcinia mangostana* L.	Induction of apoptosis. Inhibition of cell proliferation. Modulation of the PI3K/Akt/mTOR pathway. Inhibition of cell migration and invasion.	Breast, colorectal, lung, liver, glioma, and hematological cancers.	Low bioavailability due to rapid metabolism and elimination. Limited pharmacokinetics. A mandatory requirement for clinical viability is the development of complex formulations.	[54]
	Gambogic acid	*Garcinia hanburyi* Hook f.	Stimuli-responsive release. Induction of apoptosis and oxidative stress. Inhibition of HSP90.	Breast cancer.	Low bioavailability due to rapid metabolism and elimination. Limited pharmacokinetics, with low effective plasma concentrations. Systemic toxicity and adverse effects. Possible nephrotoxicity at high doses. A mandatory requirement for clinical viability is the development of complex formulations.	[55]
	Garcinone E	*Garcinia mangostana* L.	Inhibition of autophagic flux. Inhibition of cancer cell proliferation.	Nasopharyngeal cancer.	Low bioavailability due to rapid metabolism and elimination. Limited pharmacokinetics and low plasma concentrations. Possible systemic toxicity and adverse effects. The development of advanced delivery systems is essential to improve its stability.	[56]
Carotenoids	Lycopene	Tomato, watermelon, papaya, pink guava, plum, red bell pepper, carrot, pink grapefruit, asparagus, and red cabbage.	Antioxidant activity. Antiproliferative effect. Modulation of gene expression. Induction of apoptosis.	Prostate, breast, colon, esophageal, liver, ovarian, oral cavity, and lung cancers.	Low bioavailability, influenced by its lipophilic nature and the food matrix. Dose and treatment duration have not been established in humans.	[57,58,59]
	β-Carotene	Carrot, papaya, mango, *Cucurbita pepo*, spinach, broccoli, and lettuce.	Antioxidant activity. Inhibition of cancer cell proliferation. Modulation of gene expression.	Lung, breast, colon, stomach, prostate, bladder, head and neck, ovarian, skin, and hematologic cancers.	Low bioavailability, influenced by the dietary matrix and variability in absorption. Dose and treatment duration have not been established in humans. Intake above 20 mg/day in smokers may increase the risk of adverse effects, particularly lung cancer.	[60,61,62,63]
	Lutein	Spinach, carrot, yellow corn, peppers, *Xenostegia tridentata*, eggs.	Antioxidant activity. Inhibition of cancer cell proliferation and migration. Promotion of apoptosis through inhibition of the PI3K/Akt pathway. Modulation of gene expression.	Breast, prostate, liver, stomach, lung, cervical, bladder, ovarian, testicular, head and neck, and esophageal cancers.	Low bioavailability, influenced by its lipophilic nature. Optimal treatment dose and duration are not established in humans.	[64,65,66,67,68]
	Zeaxanthin	Spinach, carrots, yellow corn, peppers, egg yolk, microalgae, saffron, and seaweeds.	Antioxidant activity. Inhibition of cancer cell proliferation. Modulation of gene expression.	Ocular, prostate, colon, breast, and skin cancers.	Low bioavailability, influenced by its lipophilic nature.	[69,70]
	Astaxanthin	*Haematococcus pluvialis*, shrimp, euphausiids, crabs, salmon, and trout.	Antioxidant activity. Inhibition of cancer cell proliferation at concentrations between 150 and 200 μM (in vitro). Modulation of gene expression (caspase-3, PARP, p-p38, p-JNK, and p-ERK1/2). Induction of apoptosis through the downregulation of anti-apoptotic proteins (Bcl-2, p-Bad, survivin) and the upregulation of proapoptotic proteins (Bax/Bad and PARP).	Colorectal, prostate, breast, gastric, skin, and lung cancers.	Low bioavailability, conditioned by its lipophilic nature. Optimal human dose and treatment duration are not established. More comprehensive in vivo studies are required to validate efficacy and safety in human populations.	[71,72,73,74,75]
Terpenes	Limonene	Lime, lemon, grapefruit, orange, tangerine, mint, cannabis, pine needles, and turpentine.	Inhibition of cell proliferation through the PI3K/Akt pathway. Induction of apoptosis through Bcl-2 modulation. Modulation of multiple signaling pathways. Antioxidant activity. Antitumor activity.	Colon, lung, breast, skin, stomach, liver, lymphatic system, and bladder cancers.	Low bioavailability due to rapid metabolism. Possible adverse effects associated with high doses. Toxicity observed at doses above 1200–1600 mg/m^2^ per administration.	[76,77,78,79,80]
	Betulin	*Betula pendula*, *Betula pubescens*, *Ziziphus* spp., *Syzygium* spp.	Induction of apoptosis. Inhibition of angiogenesis. Inhibition of cell proliferation. Modulation of multiple signaling pathways. Antioxidant activity. Inhibition of carcinogenesis and metastasis.	Liver, colorectal, lung, prostate, skin, breast, head and neck, pediatric, brain, soft-tissue, bone, hematologic, lymphatic, and cervical cancers.	Low water solubility. Optimal dose has not been established for humans. May exhibit cytotoxic effects in healthy cells at high concentrations.	[81,82,83,84,85]
	Ursolic Acid	*Ocimum sanctum* L., *apple*, *Eriobotrya japonica*, *Sambucus chinensis*, *pear*, *Lavandula angustifolia*, *olive*, *rosemary*, *Punica granatum*, *Lychnis floscuculi*, *Calluna vulgaris*.	Induction of apoptosis. Inhibition of cell proliferation. Antioxidant activity. Reduction in inflammation and downregulation of pro-inflammatory gene expression. Inhibition of angiogenesis. Inhibition of metastasis through modulation of epithelial–mesenchymal transition (EMT). Modulation of NF-κB, JAK/STAT, PI3K/Akt/mTOR, MAPK, PLK1, IKK/NF-κB, and BRAF/ERK signaling pathways.	Hematologic and bone marrow cancers, hepatic, colorectal, breast, ovarian, lung, prostate, gastrointestinal, and skin cancers.	Low water solubility, which limits absorption. Optimal human dose has not been established. May exhibit cytotoxicity in healthy cells at high concentrations.	[86,87,88,89,90]
	β-Caryophyllene	*Syzygium aromaticum* (*clove*), *hops*, *rosemary*, *Piper nigrum*, *Cannabis sativa*, *Commiphora gileadensis*, *copaiba*, *Spondias pinnata*, *Pimpinella kotschyana*, and *Cananga odorata*.	Inhibition of cell growth and proliferation. Anti-inflammatory activity. Induction of apoptosis. Inhibition of angiogenesis. Activation of the CB2 cannabinoid receptor.	Hematologic and bone marrow cancers, prostate, breast, colorectal, skin, lymphatic system, ovarian, oral cavity, liver, pancreatic, bone, head and neck, and bladder cancers.	Low bioavailability due to rapid metabolism. Optimal human dose has not been established.	[91,92,93]
	Geraniol	*Palmarosa* (*Cymbopogon martinii*), *Pelargonium graveolens*, *Zingiber officinale*, *turmeric*, *lemongrass*, *citronella*, *Rosaceae species*, *lavender*, *citronella*.	Induction of apoptosis. Inhibition of cell proliferation. Antioxidant activity. Modulation of the Tyk2–STAT1/3 pathway. Inhibition of cancer cell proliferation.	Hematologic and bone marrow cancers, lung, colon, breast, central nervous system, prostate, skin, liver, kidney, pancreatic, endometrial, ovarian, and gastric cancers.	Low bioavailability due to rapid metabolism. Optimal human dose has not been established.	[94,95,96,97]
Alkaloids	Vinblastine	*Catharanthus roseus*	Induction of apoptosis. Inhibition of cell proliferation. Antioxidant activity.	Testicular, breast, lung, uterine, soft-tissue, bladder, central nervous system, gastric, and kidney cancers.	Grade III toxicity. Neurotoxicity. Multidrug resistance (MDR). May cause leukopenia, mucositis (oral ulcers), nausea, and pain as frequent adverse effects.	[98,99,100,101]
	Vincristine	*Catharanthus roseus*	Induction of apoptosis. Inhibition of cell proliferation. Antioxidant activity.	Hematologic and bone marrow cancers, soft-tissue cancers, bone cancer, testicular, uterine, breast, and lung cancers.	Neurotoxicity, including peripheral neuropathy. Multidrug resistance (MDR).	[98,99,101,102]
	Taxol (paclitaxel)	*Taxus brevifolia*, *Taxus wallichiana*, *Taxus baccata*, *Taxus cuspidata*, *Taxus × media* (*incl*. *cv*. *Hicksii*), *species of Cephalotaxus* and *Corylus avellana*.	Induction of apoptosis. Inhibition of angiogenesis. Mitotic arrest through microtubule stabilization. Antioxidant activity.	Breast, ovarian, lung, prostate, uterine, esophageal, and head and neck cancers.	Hematological toxicity (neutropenia, anemia, thrombocytopenia). Neurotoxicity (peripheral neuropathy). Low solubility, which limits its formulation. Multidrug resistance (MDR). Common side effects: hair loss, muscle pain, and allergic reactions.	[103,104,105,106]
	Colchicine	*Colchicum autumnale*, *Gloriosa superba* L.	Inhibition of inflammation. Inhibition of angiogenesis. Induction of apoptosis. Inhibition of cancer cell migration, invasion, and adhesion. Antioxidant activity.	Breast, prostate, colorectal, head and neck, esophageal, gastric, liver, pancreatic, lung, skin, cervical, endometrial, ovarian, bladder, kidney, brain, and hypopharyngeal cancers.	Low bioavailability. Multiorgan toxicity. Very narrow therapeutic window, complicating clinical use. Potential for significant cytotoxicity, even in healthy cells.	[107,108,109,110,111,112]
	Camptothecin	*Camptotheca acuminata* Decne.	Inhibition of DNA topoisomerase I. Induction of DNA strand breaks during replication. Cell cycle arrest at the S phase. Genomic damage–dependent induction of apoptosis.	Colorectal, lung, ovarian, cervical, breast, liver, gastric, pancreatic cancers, and advanced solid tumors.	Low aqueous solubility. Instability of the lactone form at physiological pH. Rapid conversion to the inactive carboxylate form. High systemic toxicity and a narrow therapeutic window limit its direct clinical use. Semisynthetic derivatives (irinotecan and topotecan) have been approved for clinical use to overcome these limitations. At the same time, nanoformulation-based delivery systems (e.g., polymeric nanoparticles, liposomes, and biomimetic carriers) are actively investigated to improve stability, bioavailability, and therapeutic index.	[113,114,115]
	Berberine	*Mahonia chinensis*, *Mahonia bealei* (*Fort*.) *Carr*., *Phellodendron chinense Schneid*., *Coptidis chinensis Franch*.	Inhibition of cell proliferation. Inhibition of angiogenesis. Modulation of multiple signaling pathways. Inhibition of cancer cell invasion and metastasis. Antioxidant activity.	Blood and bone marrow cancers, breast, lung, gastric, liver, colorectal, prostate, cervical, and ovarian cancers.	Low bioavailability due to rapid metabolism. Optimal human dosage not yet established. Poor absorption because of its hydrophilic nature.	[116,117,118,119,120]
Emerging compounds (other phytoconstituents with growing oncological relevance)	Apigenin	Oranges, onion, quinoa, basil, *Anethum graveolens*, *Petroselinum crispum*, *Coriandrum sativum*, *Apium graveolens*, *Mentha* spp., *Salvia plebeia* R.Br., *Matricaria chamomilla*, *Thymus vulgaris*, *Origanum vulgare*, *Chrysanthemum morifolium*, tea, beer, and wine.	Inhibition of cell proliferation. Induction of apoptosis. Inhibition of angiogenesis. Inhibition of cell growth and survival. Antioxidant activity.	Breast, prostate, ovarian, colon, lung, oral cavity, liver, gastric, brain, skin, and bladder cancers.	Low bioavailability due to limited metabolism and absorption. Potential interactions with other drugs, particularly anticoagulants. Hydrophobic nature, reducing systemic availability.	[121,122,123,124]
	Fisetin	Strawberries, kiwifruit, grapes, onion, garlic, peppers, *Hedyotis diffusa* Willd., *Nelumbo nucifera*, *Diospyros kaki*, apple, peach, cucumber, and nuts.	Inhibition of cell proliferation. Induction of apoptosis. Inhibition of inflammation. Antioxidant activity. Inhibition of angiogenesis.	Breast, prostate, lung, colon, brain, head and neck, bladder, laryngeal, pancreatic, kidney, liver, biliary tract, gastric, skin, oral cavity, bone marrow, ovarian, cervical, endometrial, bone, lymphatic system, and thyroid cancers.	Low bioavailability due to rapid metabolism. Potential interactions with other medications, particularly anticoagulants.	[125,126,127,128,129]
	Ginsenoside Rg3	*Panax bipinnotifidus*, *Panax elegantior*, *Panax ginseng*, *Panax japonicus*, *Panax major*, *Panax notoginseng*, *Panax omeiensis*, *Panax pseudoginseng*, *Panax quinquefolius*, *Panax sikkimensis*, *Panax sinensis*, *Panax stipuleanatus*, *Panax trifolius*, *Panax vietnamensis*, *Panax wangianus*, *Panax zingiberenensis*	Inhibition of cell proliferation. Induction of apoptosis. Inhibition of angiogenesis. Antioxidant activity.	Lung, gastric, skin, liver, breast, colon, and cervical cancers.	Low bioavailability due to limited absorption and metabolism. Hydrophobic compound, reducing systemic availability. Possible interactions with other medications, especially anticoagulants. May cause mild to moderate gastrointestinal effects.	[56,130,131,132,133,134]
	Luteolin	Chocolate, broccoli, Brussels sprouts, onion, chrysanthemum, celery, carrot, pepper, rosemary, parsley, chicory, spinach, lemon, mint, oregano, artichoke, green tea, and rooibos tea.	Inhibition of cell proliferation. Induction of apoptosis. Inhibition of inflammation. Antioxidant activity.	Breast, prostate, lung, colorectal, ovarian, skin, oral cavity, pancreatic, liver, kidney, cervical, esophageal, bladder, gastric, bone, brain, thyroid, and lymphatic system cancers.	Low bioavailability affecting absorption. Potential interactions with other medications, especially anticoagulants. Toxicity at high doses. Hydrophobic compound with limited absorption. Rapid metabolism reduces biological efficacy.	[135,136,137,138,139]
	Epigallocatechin-3-gallate (EGCG)	*Camellia sinensis*	Inhibition of cell proliferation. Induction of apoptosis. Inhibition of inflammation. Antioxidant activity. Inhibition of angiogenesis.	Colorectal, breast, lung, ovarian, endometrial, gastric, glial, and liver cancers.	Low bioavailability, limiting absorption and systemic efficacy. Possible interactions with other medications, especially anticoagulants. Preclinical toxicity reported in animal models and in vitro studies at high concentrations.	[140,141,142,143,144]
	Honokiol	*Magnolia officinalis*, *Magnolia obovate*	Inhibition of cell proliferation. Induction of apoptosis. Inhibition of inflammation. Inhibition of angiogenesis. Antioxidant activity.	Colon, skin, bone, nasopharyngeal, tongue, liver, breast, prostate, ovarian, lung, gastrointestinal, and brain cancers.	Limited studies on drug–drug interactions. May cause gastrointestinal effects. Hydrophobic compound with limited absorption. Optimal human dosage not yet established.	[145,146,147,148,149]
	Sulforaphane	Broccoli, kale, and cauliflower.	Inhibition of cell proliferation. Induction of apoptosis. Inhibition of inflammation. Antioxidant activity.	Breast, prostate, bladder, gastrointestinal, brain, gynecological, skin, lung, kidney, and pancreatic cancers.	Low bioavailability, affecting systemic absorption. Possible interactions with other medications. May cause gastrointestinal effects in some individuals.	[150,151,152,153,154]
	PEITC (Phenethyl isothiocyanate)	Watercress, broccoli, Brussels sprouts.	Inhibition of cell proliferation. Induction of apoptosis. Inhibition of inflammation. Antioxidant activity.	Gastric and colon cancers.	Low bioavailability. Hormetic effect. Low water solubility. Rapid metabolism.	[155]
	BITC (Benzyl isothiocyanate)	Broccoli and mustard.	Induction of apoptosis. Inhibition of angiogenesis. Inhibition of metastasis. Enzymatic modulation.	Breast, hematological, lung, oral, and head and neck cancers.	Uncertain pharmacokinetics in humans. Low stability and bioavailability. Rapid elimination. A mandatory requirement for clinical viability is the development of complex formulations.	[156]

**Table 2:** Critical comparison of innovative delivery platforms for bioactive compounds.

Type of Platform	Bioactive Compounds Evaluated	Delivery Mechanism or Principle	Main Advantages	Technical Limitations and Challenges	Validation Phase/Scientific Evidence	Reference
Lipid nanoparticles	Ellagic acid, curcumin, quercetin, resveratrol, lutein, zeaxanthin, astaxanthin, limonene, apigenin, berberine, and vincristine.	Composed of solid or partially liquid lipids that are biocompatible and biodegradable. They can exist as solid lipid nanoparticles (SLN) or nanostructured lipid carriers (NLC), which provide high affinity for lipophilic compounds. Drug release occurs mainly through diffusion and, in some systems, through erosion of the lipid matrix.	Biocompatibility and biodegradability. High capacity to encapsulate lipophilic compounds. Improved oral bioavailability and protection against chemical/photo-oxidative degradation.	Limited physical and chemical stability (recrystallization, drug expulsion). Challenges in scaling up production. Dependence on surfactants and excipients that may cause irritation or cumulative toxicity.	Extensive preclinical evidence in in vitro and in vivo models. Phase I and II clinical trials available for several lipid nanoparticle-based formulations. Some lipid-based formulations (not necessarily pure SLN/NLC) have been approved for clinical application, supporting the safety of this approach.	[121,157,158,159,160,161,162,163,164,165,166]
Polymeric nanoparticles	Curcumin, quercetin, resveratrol, ellagic acid, berberine, ginsenoside Rg3, and epigallocatechin-3-gallate.	Synthesized from biodegradable polymers such as polylactic acid (PLA) or polylactic-co-glycolic acid (PLGA). They can be engineered to provide controlled drug release in response to specific stimuli.	Flexibility in synthesis and surface modification. Ability to encapsulate a wide variety of compounds. Controlled drug release.	Difficulty in precisely controlling drug release. Potential issues related to biodegradability and biocompatibility. Higher production costs compared with conventional formulations.	Preclinical studies have evaluated their potential for drug delivery. Some formulations are currently in clinical development phases.	[167,168,169,170,171,172,173]
Liposomes	Ellagic acid, curcumin, quercetin, resveratrol, and berberine.	Spherical vesicles composed of lipid bilayers that encapsulate hydrophilic or lipophilic drugs. Drug release occurs through diffusion across the lipid bilayer or via endocytosis.	Biocompatibility and biodegradability. Ability to encapsulate both hydrophilic and lipophilic drugs. Improved bioavailability and reduced toxicity.	Limited physical and chemical stability. Challenges in scaling up production. Potential long-term toxicity issues.	Several liposomal products have been approved for clinical use, such as Doxil^®^ (liposomal doxorubicin) and Onivyde^®^ (liposomal irinotecan). Other formulations are currently under in vitro and in vivo investigation.	[174,175,176,177,178]
Micelles	Curcumin, quercetin, resveratrol, ellagic acid, berberine, and ginsenoside Rg3.	Spherical structures composed of amphiphilic molecules that self-assemble in aqueous media. Lipophilic drugs are encapsulated within the hydrophobic core of the micelle.	High loading capacity for lipophilic drugs. Stability in aqueous environments. Easy surface modification for targeted delivery.	Limited stability in biological environments. Difficulty in controlling drug release. Potential toxicity issues due to the presence of surfactants.	In vitro and in vivo studies have demonstrated inhibition of cancer cell growth. Ongoing preclinical and clinical studies are evaluating the efficacy and safety of micelles as drug delivery platforms.	[172,179,180,181,182]
Nanoemulsions	Curcumin, quercetin, resveratrol, ellagic acid, lutein, zeaxanthin, astaxanthin, and limonene.	Oil-in-water dispersions stabilized by surfactants. Lipophilic drugs are dissolved in the oil droplets.	High loading capacity for lipophilic drugs. Good physical and chemical stability. Easy scaling-up and production.	Limited physical stability. Difficulty in controlling droplet size. Potential toxicity issues due to the presence of surfactants.	In vitro and in vivo studies show that they can prevent the migration of cancer cells. Ongoing preclinical and clinical studies are evaluating the efficacy and safety of nanoemulsions as drug delivery platforms, particularly for dermatological and nutritional applications.	[183,184,185,186,187,188,189,190]
Hydrogels	Ellagic acid, curcumin, quercetin, resveratrol, berberine, and ginsenoside Rg3.	Three-dimensional networks of hydrophilic polymers capable of absorbing and retaining large amounts of water. Drug release occurs through diffusion across the hydrogel matrix.	Biocompatibility and biodegradability. Ability to incorporate a wide variety of bioactive compounds. Controlled drug release.	Limited physical and chemical stability. Difficulty in achieving precise drug release control. Potential issues related to biocompatibility and biodegradability.	Preclinical and clinical studies have demonstrated their efficacy and safety in drug delivery. Some formulations have been approved for clinical use.	[191,192,193,194,195,196]
Stimuli-responsive smart systems	Ellagic acid, curcumin, quercetin, resveratrol, and berberine.	Hydrogels that respond to environmental changes such as pH, temperature, light, redox conditions, and more. Drug release occurs in response to these stimuli, which alter the structure and permeability of the hydrogel.	Targeted and controlled drug release in response to specific stimuli. Improved drug bioavailability and efficacy. Reduction in side effects.	Complexity in synthesis and design. Difficulty in achieving precise and controlled responsiveness to stimuli. Potential issues related to stability and scalability.	Preclinical studies have evaluated their potential in drug delivery. Some formulations are currently in clinical development phases.	[197,198,199,200,201]
Hybrid systems	Ellagic acid, curcumin, quercetin, resveratrol, berberine, and ginsenoside Rg3.	These systems combine different technologies—such as liposomes, micelles, and nanoemulsions—to create customized delivery platforms. They may incorporate external stimuli such as pH, temperature, or light to control the release of the bioactive compound.	Greater flexibility and customization in the delivery of bioactive compounds. Improved bioavailability and stability of the compounds. Ability to incorporate multiple bioactive compounds into a single system.	Complexity in formulation and scale-up. Potential issues related to stability and biodegradability. Regulatory challenges and approval requirements from health authorities.	Preclinical and clinical studies are underway to evaluate the efficacy and safety of these systems.	[178,202,203,204,205,206]
Emerging platforms	Curcumin, quercetin, resveratrol, ellagic acid, lutein, zeaxanthin, astaxanthin, and limonene.	Isotropic mixtures of lipids, surfactants, and cosolvents that generate fine oil-in-water (O/W) emulsions upon exposure to gastrointestinal fluids, thereby improving the solubility and bioavailability of lipophilic drugs.	Significant improvement in the oral bioavailability of poorly water-soluble drugs. Enhanced stability of drug molecules. Possibility of delivering the final product in various pharmaceutical dosage forms.	Difficulty in predicting drug precipitation within the gastrointestinal tract. Require careful formulation optimization. Potential long-term physical and chemical stability issues.	Preclinical and some clinical studies have demonstrated their potential to enhance the bioavailability of poorly soluble drugs. Several formulations are currently in clinical development phases.	[207,208,209,210,211,212,213,214]

**Table 3:** Preclinical, early clinical, and translational evidence of bioactive compounds formulated for oncological applications.

Bioactive Compounds	Drug Delivery Technological Platform	Study Phase/Target Cancer	Biomedical Relevance	Reference
Quercetin	Plasma-derived exosomes (biomimetic).	Derived exosomes (biomimetic) In vitro and in vivo (murine glioma model).	The Que/mAb GAP43-Exo system significantly reduced ROS production and upregulated the expression of four key antioxidant enzymes (NQO1, HO-1, SOD1, and GPx1) through activation of the Nrf2 signaling pathway.	[219]
	Gold nanoparticles (AuNPs).	In vitro (A549 and HeLa cancer cell lines).	The formulation exhibited a half-maximal inhibitory concentration (IC50) of 45.23 µg/mL in A549 cells and 38.42 µg/mL in HeLa cells.	[220]
Fisetin	Senolytic nanomicelles/polymeric nanomicelles.	In vivo (MCF-7 breast cancer model).	The formulation achieved a drug loading efficiency of 82.50 ± 1.78%, with an average nanoparticle diameter of 103.2 ± 6.1 nm, resulting in a six-fold increase in bioavailability compared with free fisetin.	[221]
Kaempferol	Niosomal nanoparticles.	In vitro (MCF-7 breast cancer cells).	The formulation achieved an IC50 value of 0.0873 µM in MCF-7 cells, inducing 64% apoptosis, with no significant toxicity observed in healthy cells.	[222]
	Metal–organic frameworks (Kae–Fe photothermal nanoparticle platform).	In vitro and in vivo (4T1 TNBC model).	The photothermal Kae–Fe nanoparticle platform achieved 87% cell death in 4T1 cells, with a photothermal conversion efficiency of 91.0%.	[223]
Apigenin	Bio-responsive Metal–Organic Framework Nanoplatform.	In vitro and in vivo (4T1 TNBC model).	The ZDAP system (ZIF-90/AP/DOX) enabled an effective triple therapy combining chemotherapy, photothermal therapy, and metabolic lactate/ATP depletion.	[224]
Luteolin	Self-assembling supramolecular hydrogel.	In vivo (pre-colorectal cancer model).	The hydrogel protected luteolin from gastric degradation, enabling the delivery of an effective therapeutic dose to the site of inflammation in the colon.	[225]
Epigallocatechin gallate (EGCG)	NanoCubeSpray delivery system.	Ex vivo and in vivo (oral submucous fibrosis model).	The formulation reduced type I collagen deposition and TGF-β1 expression, thereby preventing fibrosis and restoring antioxidant levels.	[226]
Catechin	Gold–catechin nanohybrids.	In vitro (breast cancer model).	The nanohybrid system enabled a combined therapeutic strategy involving photothermal therapy (laser-induced heating) and natural chemotherapy.	[227]
Hesperidin	Mucoadhesive biopolymeric nanoparticles.	In vitro (MDA-MB-231 breast cancer cells).	The formulation exhibited superior qualitative efficacy in terms of antioxidant activity, apoptosis induction, and cell cycle arrest compared with the free compound.	[48]
Naringenin	Biomimetic red blood cell membrane–coated nanoparticles.	In vitro (HepG2 hepatocellular carcinoma cells).	The NARNPs reduced the IC50 to 1.6 µg/mL compared with 22.32 µg/mL for free naringenin and induced late apoptosis in 56.1% of cancer cells.	[49]
Eriodictyol	Mesoporous nanocubes.	In vitro and in vivo (osteosarcoma model).	The nanocube formulation markedly enhanced ferroptosis and cisplatin sensitivity without evident systemic toxicity.	[50]
Didymin	Direct administration of the compound.	In vitro and in vivo (AGS and HGC-27 gastric cancer models).	Didymin significantly reduced cell viability and tumor volume in vivo (*p* < 0.01) and increased apoptosis through downregulation of p-PI3K and p-Akt.	[51]
Poncirin	Direct administration of the compound.	In vitro (HeLa cervical cancer cells).	The compound was evaluated over a concentration range of 5–160 µM, resulting in a substantial reduction in cell growth and a significant increase in caspase activity and apoptosis induction.	[52]
α-mangostin	Polymeric nanoparticles.	In vitro and in vivo (MCF-7 breast cancer model).	The nanoformulation achieved a 17.43% reduction in tumor volume by day 14, exhibiting a threefold higher efficacy compared with free α-mangostin.	[53]
γ-mangostin	Biosynthesized silver nanoparticles.	In vitro (MCF-7 breast cancer cells).	Effective cytotoxicity, morphological degeneration, and significant antiproliferative activity were observed over a concentration range of 5–70 µg/mL.	[54]
Gambogic acid	Mesoporous polydopamine nanoparticles.	In vitro and in vivo (TNBC model).	The nanoparticles achieved a drug loading capacity of 75.96%, enabling significant inhibition of tumor growth through combination with photothermal therapy.	[55]
Garcinone E	Subcutaneous injection.	In vitro and in vivo (HK1, HONE1, and S18 nasopharyngeal carcinoma models).	IC50 values ranging from 4.65 to 8.83 µmol/L (72 h) were observed, along with significant inhibition of tumor growth in vivo at a dose of 35 mg/kg administered every three days.	[228]
Resveratrol	Milk-derived exosomes.	In vitro and in vivo (MCF-7 and MDA-MB-231 breast cancer cells and mammary tissue).	Peak concentrations of 41 ± 15 nM (curcumin, CUR) and 300 ± 80 nM (resveratrol, RSV) were achieved in mammary tissue within 6 min, resulting in potent antiproliferative activity at nanomolar concentrations ineffective for the free compounds.	[229]
Curcumin	Camel milk–derived exosomes.	In vitro (A549 and A549TR lung cancer cells).	The formulation exhibited a drug loading efficiency of 20%, resulting in significantly enhanced cytotoxicity in both drug-sensitive and drug-resistant cells compared with free curcumin.	[230]
	Biocompatible Mg/PLGA/CHI micromotors.	In vitro (HepG2 hepatocellular carcinoma cells).	Cellular uptake was enhanced 1.5-fold, leading to a 30% reduction in cell proliferation at a concentration of 1 mg/mL compared with the passive group.	[231]
Podophyllotoxin	Macrocyclic resin glycosides.	In vitro (MCF-7 breast cancer cells); clinically approved derivatives: etoposide (lung and testicular cancer) and teniposide (FDA-approved).	The combination of resin glycosides (1–50 µM) with sublethal doses of vinblastine and podophyllotoxin (0.003 µM) enhanced cytotoxicity, while the glycosides alone showed no toxicity (IC50 > 25 µM).	[232]
Viscumin	Surface-imprinted polymer-based nanobiosensor.	In silico.	A 9-mer epitope (QQTTGEEYF) was identified, with a molecular weight of 1102.1 Da, an isoelectric point of 3.79, and a prediction accuracy greater than 50%.	[233]
Lectin	Glycan-targeted biotherapeutic agent.	In vitro (A549, H460, and H1299 lung cancer cell lines); clinically used formulation: Iscador (Swissmedic-approved) for breast, colorectal, lung, gastric, and melanoma cancers.	The combined treatment reduced cell viability to 40% (compared with 70–90% for monotherapies) and inhibited invasion and migration to levels below 20%.	[234]
Ellagic acid	Metal–organic framework nanoplatform (CS/NP).	In vitro and in vivo (MCF-7/Adr, MCF-7, and 4T1 breast cancer models).	The nanoplatform significantly reversed chemoresistance by inducing cuproptosis and effectively eliminating tumors of approximately 250 mm^3^.	[235]
Camptothecin	Nanoliposomal formulation of irinotecan.	Phase III (lung cancer); FDA-approved derivatives: irinotecan (colorectal cancer) and topotecan (ovarian and lung cancer).	The liposomal formulation doubled the objective response rate (44.1% vs. 21.6%) and significantly reduced grade ≥3 treatment-related adverse events (42.0% vs. 83.4%) compared with topotecan.	[236,237,238]
	pH-responsive polymeric nanoparticles with sustained release.	In vitro and in vivo (A431 epidermoid carcinoma model).	The nanoformulation achieved a five-fold increase in bioavailability, an IC50 value of 3 µg/mL, and an 80% survival rate in animal models.	[239]
Vinblastine	Panel of isogenic chemoresistant cancer cell models.	In vitro (bladder cancer models).	The study identified specific resistance mechanisms, including mutations in PIK3CA, KRAS, and FGFR3, as well as alterations in ABCB1 and SLC3A1 genes, enabling the development of personalized therapeutic strategies.	[240]
Vincristine	PCL/C-dots–based double emulsion nanotheranostic system.	In vitro (hepatoblastoma and hepatocellular carcinoma models).	The PCL-based nanotheranostic platform (≈200 nm) demonstrated superior antitumor efficacy compared with the free drug, achieving enhanced cell growth inhibition and optimal colloidal stability (PDI < 0.5).	[241]
Berberine	Targeted polymeric nanocarriers.	In vitro and in vivo (lung cancer models).	The targeted nanocarriers demonstrated improved bioavailability and reduced systemic toxicity.	[242]
Colchicine	Enzyme-transformable polymeric polymersomes.	In vitro and in vivo (231/LM2, 4T1, and HT1080 cancer models).	The transformable polymersomes exhibited a 35-fold higher tumor retention than the non-transformable counterpart at 48 h, with 60–80% of mice remaining recurrence-free after surgery.	[243]
Paclitaxel	Biohybrid neutrobots.	In vitro and in vivo (GL261 glioma model).	Paclitaxel-loaded neutrobots doubled the median survival of mice (from 18 to 41 days) and achieved a four-fold increase in brain drug accumulation compared with conventional delivery methods.	[244]
	Core–shell nanoparticles.	In vitro (4T1 TNBC model).	The nanocapsules (≈80 nm) exhibited an exceptionally high drug loading capacity (750%) and enabled pH-responsive release of paclitaxel, effectively inhibiting 4T1 cells for up to 48 h.	[245]
β-Caryophyllene	Small-molecule drug design.	In vitro (HT-29, HCT-116, and HCT-15 colorectal cancer cell lines).	The compound exhibited an IC50 value of 2.49 µM, demonstrating markedly higher potency compared with natural β-caryophyllene.	[246]
Artemisinin	Ferroptosis-inducing nanoreactors.	In vitro and in vivo (TNBC models: 4T1 and E0771).	The 1:1 nanoassemblies induced massive ferroptosis and significant tumor regression through iron overload and glutathione (GSH) depletion in TNBC models.	[247]
Ursolic acid	Photosensitive nanoparticles.	In vitro and in vivo (MCF-7 and HepG2 cancer models); Phase I (advanced solid tumors).	Superior antitumor efficacy was achieved by combining ursolic acid with phototherapy.	[248]
Betulin	Lactoferrin-based bionanocarriers.	In vitro (MDA-MB-231 and HEp-2 cancer cell lines).	The nanoparticles enabled rapid cellular uptake within 30 min and induced potent cytotoxicity and apoptosis, significantly outperforming free betulinic acid after 24 h.	[249]
Ginsenoside Rg3	Polymeric nanoparticles.	In vitro and in vivo (4T1 TNBC model).	The nanoformulation achieved a tumor growth inhibition rate exceeding 80%, significantly enhancing survival and promoting the infiltration of cytotoxic T lymphocytes within the tumor microenvironment.	[250]
Limonene	AI-optimized hydrogel microarrays	In vitro and in vivo (PC-3 prostate cancer model).	Liposomes with a mean diameter of 36.23 nm achieved a penetration depth of 467.87 µm and a permeation rate of 41.78%, significantly inhibiting cancer cell growth and safely promoting apoptosis in vitro.	[251]
Geraniol	Targeted polymeric nano-conjugate.	In vitro, In vivo/PC-3.	The nano-conjugate significantly inhibited tumor growth in vivo with high biocompatibility and reduced cell viability through mitochondria-mediated apoptosis.	[94]
Withaferin A	Bio-targeted gold nanoparticles.	In vitro and in vivo (MDA-MB-231 and MCF-7 breast cancer models).	The nanoformulation significantly reduced tumor growth through the induction of oxidative stress and selective apoptosis.	[252]
Cucurbitacin B	Biomimetic nanoplatform based on synthetic vesicles (exosome-like).	In vitro and in vivo (gastric, colorectal, pancreatic, breast, lung, and hepatocellular carcinoma models); clinically used formulation: cucurbitacin tablets approved by the MNPA (China).	Cucurbitacin B exhibited potent inhibition of tumor growth and metastasis and has been used as a clinical adjuvant since the 1970–1980s.	[253]
Sulforaphane	Controlled-release nanoparticles.	Phase II (prostate and breast cancer); Phase I (colon cancer); in vivo (ovarian and lung cancer models); in vitro (pancreatic cancer cells).	The nanoformulation induced selective apoptosis, reduced cell viability by up to 70%, and significantly inhibited tumor volume in vivo through effective modulation of the Nrf2/Keap1 signaling pathway.	[153]
Phenethyl isothiocyanate (PEITC)	Biopolymeric microcapsules obtained by complex coacervation.	In vitro (SW48 and MKN45 colorectal cancer cell lines).	The microcapsules achieved an encapsulation efficiency of 94.2%, reduced colon cancer cell viability by 65%, and maintained stability under gastric pH conditions.	[155]
Benzyl isothiocyanate (BITC)	Stable nanoemulsions.	In vitro (MDA-MB-231 and MCF-7 breast cancer cell lines).	The nanoemulsion achieved an IC50 value of 7.8 µM in MDA-MB-231 cells, outperforming the free compound and reducing angiogenic branching points by 50%.	[156]
Allicin	Active-targeted precision nanovehicular platform.	In vitro (MCF-7 breast cancer cells).	The nanoplatform achieved an encapsulation efficiency of 86.3% and exhibited an IC50 value of approximately 20 µg/mL.	[254]
β-Carotene	Solid lipid nanovehicular platform.	In silico and in vitro (MCF-7 breast cancer cells).	The nanovehicular system achieved an encapsulation efficiency of 84%, exhibited sustained release (40.21% at pH 5.2), and showed an IC50 value of 22.82 µg/mL.	[255]
Lycopene	Self-assembled nanomicellar platform.	In vitro (MCF-7 and HepG2 cancer cell models).	The nanomicellar platform overcame gastric degradation with an efficiency of 92.6% and achieved an IC50 value of 14.58 µg/mL, supporting its potential as a targeted oral therapy with high bioavailability.	[256]
Lutein	Magnetically responsive biopolymeric core–shell nanovehicular platform.	In vitro (MCF-7 breast cancer cells).	Magnetic nanoparticles increased cytotoxicity four-fold in MCF-7 cells through magnetic targeting, demonstrating translational feasibility for site-specific and biocompatible lutein delivery.	[257]
Astaxanthin	Active-targeted precision nanovehicular platform.	In vitro (MCF-7 and MDA-MB-231 breast cancer cell lines).	The nanovehicular system achieved an IC50 value of 11.23 µg/mL in MCF-7 cells, demonstrating CD44 receptor–mediated cellular uptake and significantly enhanced therapeutic efficacy compared with free astaxanthin.	[258]
Zeaxanthin	Zein nanoparticles.	In vitro (CD8^+^ T cells, B16F10-luc2, B16F10, B16-OVA, and MC38 models); in vivo (B16F10 and MC38 tumor models, CD8^+^ T cells).	The nanoformulation markedly reduced tumor volume and potentiated anti-PD-1 immunotherapy by stabilizing the TCR complex and enhancing effector cytokine secretion in CD8^+^ T cells.	[69]
Glucose oxidase	Bioautonomous Janus nanomotors.	In vitro (HeLa, SK-Mel-103, 4T1 cancer cell lines, and patient-derived organoids, PDOs); in vivo (4T1 tumor model).	The nanomotors achieved a significant reduction in tumor volume and superior deep penetration in organoids and tumors in vivo, outperforming conventional passive nanoparticles.	[259]
Doxorubicin	Bio-intelligent polymeric nanocarriers.	In vitro and in vivo (MCF-7, HepG2, HeLa, A549, and MCF-7/ADR cancer models).	The nanocarriers achieved encapsulation efficiencies exceeding 90%, enabled pH-responsive drug release, and significantly reduced IC50 values, effectively reversing P-glycoprotein–mediated chemoresistance.	[260]

## Data Availability

No new data were created or analyzed in this study. Data sharing is not applicable to this article.

## Abbreviations

The following abbreviations are used in this manuscript:

ATG5	Autophagy-related gene 5; key regulator of autophagosome formation.
AuNPs	Gold nanoparticles.
Bad	Bcl-2 antagonist of cell death; proapoptotic protein that antagonizes Bcl-2 function.
Bad/Bax	Proapoptotic proteins of the Bcl-2 family that promote mitochondrial permeabilization.
Bax	Bcl-2 associated X protein; proapoptotic mediator of mitochondrial permeabilization.
BACH1	BTB and CNC homology 1; transcription factor involved in oxidative stress response and ferroptosis regulation.
Bcl-2	B-cell lymphoma 2; antiapoptotic protein that inhibits cell death.
BRAF/ERK	BRAF serine/threonine kinase and extracellular signal-regulated kinase; components of the MAPK pathway involved in tumor growth.
CB2	Cannabinoid receptor type 2; receptor involved in immune regulation and inflammation.
Caspase-3	Key protease in the execution phase of apoptosis.
DNA	Deoxyribonucleic acid.
Doxil^®^	Liposomal doxorubicin.
DoE	Design of Experiments; statistical method for experimental optimization.
EGCG	Epigallocatechin-3-gallate; major bioactive catechin from green tea.
EMT	Epithelial–Mesenchymal Transition; process associated with invasion and metastasis.
ERK1/2	Extracellular signal-regulated kinases 1 and 2; regulators of proliferation, differentiation, and survival.
FDA	U.S. Food and Drug Administration.
FGFR3	Fibroblast growth factor receptor 3.
GMP	Good Manufacturing Practice.
GPX4	Glutathione peroxidase 4; enzyme that protects cells from lipid peroxidation and ferroptosis.
GSH	Reduced glutathione; major intracellular antioxidant.
HO-1	Heme oxygenase 1; antioxidant and cytoprotective enzyme.
HSP90	Heat shock protein 90; molecular chaperone involved in cancer cell survival.
IAP	Inhibitor of Apoptosis Proteins; family of apoptosis-suppressing proteins.
IC50	Half-maximal inhibitory concentration.
IKK	IκB kinase; complex that activates NF-κB by phosphorylating IκB.
JAK/STAT	Janus kinase/Signal transducer and activator of transcription pathway.
JNK	c-Jun N-terminal kinase; kinase involved in stress response, apoptosis, and proliferation.
Keap1	Kelch-like ECH-associated protein 1; negative regulator of Nrf2 signaling.
LC3	Microtubule-associated protein 1A/1B-light chain 3; marker of autophagy.
MAPK	Mitogen-activated protein kinase; pathway regulating proliferation, differentiation, and stress responses.
MDR	Multidrug Resistance; resistance to multiple chemotherapeutic agents.
MOF	Metal–organic framework; porous coordination material used in drug delivery.
mTOR	Mammalian target of rapamycin; key regulator of cell growth, metabolism, and survival.
NF-κB	Nuclear factor kappa B; regulator of inflammation and cell survival.
NLC	Nanostructured lipid carriers.
NP	Nanoparticles; nanometer-scale carriers used for drug delivery.
NQO1	NAD(P)H quinone dehydrogenase 1; enzyme involved in cellular redox balance.
Nrf2	Nuclear factor erythroid 2–related factor 2; regulator of antioxidant and cytoprotective responses.
O/W	Oil-in-water emulsion.
Onivyde^®^	Liposomal irinotecan.
p-Bad	Phosphorylated Bad; proapoptotic protein inactivated by phosphorylation.
p-ERK1/2	Phosphorylated ERK1/2; regulators of proliferation and differentiation.
pH	Potential of hydrogen; measure of acidity or basicity.
p-JNK	Phosphorylated c-Jun N-terminal kinase; component of the MAPK pathway involved in apoptosis and stress response.
p-p38	Phosphorylated p38 kinase; MAPK member associated with stress and apoptosis.
p38 MAPK	p38 mitogen-activated protein kinase; regulator of inflammation and stress signaling.
PARP	Poly(ADP-ribose) polymerase; enzyme involved in DNA repair and cell death signaling.
PDI	Polydispersity index; measure of particle size distribution.
PI3K/Akt	Phosphatidylinositol 3-kinase/Akt signaling pathway.
PLGA	Poly(lactic-co-glycolic acid); biodegradable copolymer.
PLA	Polylactic acid; biodegradable polymer.
PLK1	Polo-like kinase 1; regulator of mitosis and cell cycle progression.
PDOs	Patient-derived organoids.
REDOX	Reduction–oxidation reactions; key cellular metabolic processes.
ROS	Reactive oxygen species; chemically reactive molecules involved in oxidative stress.
SLN	Solid lipid nanoparticles.
SOD1	Superoxide dismutase 1; enzyme responsible for reactive oxygen species detoxification.
STAT1/3	Signal transducer and activator of transcription 1 and 3; transcription factors involved in inflammation and apoptosis.
Survivin	Inhibitor of apoptosis protein (IAP family) and mitosis regulator.
TNBC	Triple-negative breast cancer.
Tyk2	Tyrosine kinase 2; JAK family member involved in immune and inflammatory signaling.
ZIF	Zeolitic imidazolate framework; subclass of metal–organic frameworks.
